# Sex Differences in Bench Press Muscle Activation With and Without Pre‐Exhaustion of Triceps Brachii

**DOI:** 10.1002/ejsc.70290

**Published:** 2026-11-07

**Authors:** M. Charlotte Olsson, Jenny Fälth, August Ahlebrand, Sofia Ryman Augustsson

**Affiliations:** ^1^ Department of Environmental and Biosciences School of Business, Innovation and Sustainability Halmstad University Halmstad Sweden; ^2^ Halmstad University Halmstad Sweden; ^3^ Department of Sport Science Faculty of Social Sciences Linnaeus University Kalmar Sweden

**Keywords:** deltoideus anterior, electromyography, pectoralis major, resistance training, women

## Abstract

The aim of this study was to investigate possible sex‐differences in upper‐body relative strength and the magnitude of muscle activity during the bench press exercise with and without pre‐exhaustion (PreEx) of triceps brachii (TB) before the bench press. Resistance trained women (*n* = 15) and men (*n* = 15) were assigned to a crossover design where the order of PreEx was block randomized. Each participant completed two 10RM bench press, one without PreEx and one which followed immediately after PreEx to failure with 5 minutes rest in between. Before bench press electromyography electrodes were attached to muscles pectoralis major (PM), TB and deltoideus anterior (DA) and data were normalized to maximum voluntary isometric contraction (%MVIC). For data analyses, a mixed model ANOVA was used. Results showed that both women and men performed a similar number of repetitions in the bench press after PreEx (*p* = 0.55). A sex‐difference was found in TB muscle activation where PreEx before bench press increased TB activation in women (post‐hoc *p* = 0.02) but not in men (post‐hoc *p* = 0.36). Moreover, PM activation increased similarly in women and men (post‐hoc *p* < 0.01 for both) following PreEx, while no changes in DA activation were detected in either sex. During the bench press without PreEx, no sex‐differences in muscle activation (%MVIC) were found in any of the three muscles examined. In conclusion, in resistance‐trained women, PreEx of the TB enhances activation of both the TB and PM during the bench press, whereas in men, PreEx increases activation only in the PM.

## Key Points

- Resistance‐trained women and men can use pre‐exhaustion (PreEx) of a target muscle during multi‐joint exercises to vary training stimuli and volume. However, research on PreEx in women is limited, and it remains unclear whether sex differences exist in its effects during the bench press.
- PreEx of the triceps brachii before the bench press can effectively enhance activation of primary movers, such as the pectoralis major, in both women and men, supporting its use for promoting hypertrophy or neuromuscular adaptations. However, the distinct triceps brachii response observed in women suggests that training volume and fatigue should be carefully monitored when applying PreEx to female athletes or clients.
- Coaches and practitioners should consider that following triceps brachii fatigue, women may exhibit greater compensatory activation of this muscle during subsequent bench press performance. This may warrant different accessory training or recovery strategies compared with men.

## 1 Introduction

Resistance training has been consistently demonstrated to be effective in enhancing athletic performance and is now a standard element in conditioning programs for athletes across various disciplines (Cid‐Calfucura et al. 2023; Crowley et al. 2017; Nuñez et al. 2022; Ryman Augustsson et al. 2024). To enhance strength, power, and hypertrophy, it is recommended to vary training stimuli by adjusting factors such as exercise order, training volume, and muscle activation (Tan 1999). Thus, a range of resistance training techniques are used to augment strength and power, thereby improving overall physical performance in sports (Campos et al. 2002; Fisher et al. 2014). In addition, several of these methods are employed to optimize muscle hypertrophy, which serves as a primary objective in bodybuilding (Krzysztofik et al. 2019; Steele et al. 2019). This goal is also frequently pursued by individuals involved in recreational strength training (McQuilliam et al. 2020) as well as in specific rehabilitation protocols (De Renty et al. 2023; Sajiki‐Ito et al. 2024). One method used to vary exercise order and muscle activation is pre‐exhaustion (PreEx), which includes performing a single joint exercise to fatigue the synergist or agonist muscle, before performing a multi‐joint exercise with minimal recovery between the transition (Jones 1970). This advanced method seeks to enhance muscle activation in the prime movers of multi‐joint exercises, resulting in a greater training stimulus and training‐induced hypertrophy compared to traditional resistance training (Trindade et al. 2022). PreEx has been proposed as an effective method for overcoming strength plateaus (Ribeiro et al. 2019), particularly for advanced strength athletes. By engaging in PreEx of a muscle through a single‐joint movement, it may be possible to decrease the load required for subsequent multi‐joint exercises (Schoenfeld et al. 2021). Thus, PreEx may benefit long‐term joint health by allowing advanced strength trained athletes to use lighter loads in multi‐joint lifts, reducing joint stress after fatiguing target muscles with single‐joint exercises (Schoenfeld et al. 2021).

The desired outcome of the PreEx method is to increase neuromuscular activation in the muscle or muscles in the multi‐joint exercise that has not been pre‐exhausted during the exercise (Brennecke et al. 2009). The original concept, proposed by Jones (1970), suggests that to achieve optimal results from PreEx, exercise order should be arranged such that a single‐joint movement is performed to muscular failure, immediately followed by a multi‐joint exercise to muscular failure. Current evidence indicates that PreEx yields at most a small, context dependent effect, characterized by modest changes in training volume or region specific hypertrophy, without clear practical advantages for target muscle involvement, strength, or overall hypertrophy compared with traditional resistance training (Ribeiro et al. 2019; Trindade et al. 2022). However, most studies on PreEx, both acute and chronic, deviate from the original concept proposed by Jones (1970) and include diverse study designs. For example, existing PreEx studies (Ribeiro et al. 2019; Trindade et al. 2022) range from acute, EMG‐based designs using moderate‐to‐high loads and short but non‐immediate transitions between exercises to chronic training interventions differing in exercise order, loading, rest intervals, and hypertrophy assessment methods, highlighting considerable methodological heterogeneity relative to the original PreEx concept. Thus, the diversity of approaches and variations employed complicates the ability to draw definitive conclusions regarding the efficacy of PreEx.

PreEx research has been conducted to examine its effects on both the lower (Augustsson et al. 2003; Trindade et al. 2019) and upper extremities (Fisher et al. 2014; Soares et al. 2016). The most commonly investigated upper extremity exercise in PreEx research seems to be the bench press (Trindade et al. 2022). The bench press is one of the most commonly used resistance exercises for training upper body strength and power (Padulo et al. 2015; Travis et al. 2020) and is one component that, together with squat and deadlift, constitutes the big three and the competition elements included in powerlifting (Androulakis‐Korakakis et al. 2021; Ferland et al. 2020). The bench press targets three main muscle groups: the pectoralis major (PM), deltoideus anterior (DA), and triceps brachii (TB) (Stastny et al. 2017). A systematic review on muscle activity during the bench press exists (Stastny et al. 2017), including some articles that studied sex‐differences in muscle activation (Gołaś et al. 2017; Mausehund and Krosshaug 2023). Still, the effects of PreEx on neuromuscular activation during bench press is less abundant and had only been observed in men (Brennecke et al. 2009; Gentil et al. 2007; Gołaś et al. 2017; Guarascio et al. 2016; Soares et al. 2016). In a couple of studies, the researchers investigated PreEx of the prime mover, PM, in bench press (Brennecke et al. 2009; Gentil et al. 2007; Gołaś et al. 2017). In one study, Gentil et al. (2007) compared the effects of performing a multi‐joint exercise (chest press) before versus after a single‐joint exercise (pec deck). The results showed no significant difference in total repetitions or total work between the two exercise orders. However, muscle activation was significantly higher in the TB following PreEx of the PM muscle. No differences in muscle activity were observed for the DA or PM in the 13 men who participated (Gentil et al. 2007). Similar findings were reported by Brennecke et al. (2009) in a study of 12 men. However, in both studies, the original protocol, where a single‐joint exercise is performed to fatigue the synergist or agonist muscle prior to the bench press, was not followed. Instead, in these studies, the authors employed a fatigue protocol that involved fatiguing the same muscle (PM) in the PreEx protocol. In another study (Soares et al. 2016), the synergist muscle (TB) was fatigued through push‐downs prior to the bench press. No effect on muscle activity was observed in this study, which included only male participants.

Only Vilaça‐Alves et al. (2014) appear to have investigated sex differences in PreEx, but their protocol focused on a biceps brachii exercise prior to a front lateral pull‐down, with repetitions counted both with and without PreEx. Additionally, only Fisher et al. (2014) included women as participants in a PreEx bench press study. However, they employed a longitudinal design, did not report results separately for men and women, and did not include electromyography (EMG) measurements. To our knowledge, no study has included women in PreEx research involving EMG measurements of the target muscles in multi‐joint movements, nor has any study examined potential sex differences in muscle activation following PreEx. Hence, the muscle activation response to PreEx in the bench press for women is unknown and needs to be investigated to allow appropriate recommendations to be made. The aim of this study was to investigate possible sex‐differences in upper‐body relative strength and the magnitude of muscle activity during the bench press exercise with and without PreEx of TB before the bench press.

## 2 Materials and Methods

### 2.1 Study Design and Participants

A crossover design was employed with a total of 30 participants (15 men and 15 women). The participants were recruited via flyers distributed at a gym and through word‐of‐mouth referrals. The inclusion criteria were as follows: (1) healthy individuals, (2) aged between 20 and 40 years, and (3) with at least 1 year of resistance training experience, performing a minimum of three sessions per week, (4) the participants had to incorporate the bench press exercise into their routine at least once per month. Exclusion criteria were (1) presence of musculoskeletal injuries in the upper body. All participants received written and oral information about the project, were informed of the benefits and risks, and gave informed consent. The study followed the ethical guidelines presented in the Declaration of Helsinki (World Medical Association 2013), and was approved by the Regional Ethical Review Board in Sweden (Dnr 2013/707).

### 2.2 Experimental Design

The participants visited the testing facility on two occasions. During the first visit (Session 1), body composition was assessed, including measurements of height, body mass, and bioelectrical impedance analysis (InBody770, Biospace, South Korea). Participants arrived having fasted for the previous 3 hours. Following the body composition assessments, but prior to engaging in the resistance exercise, they were permitted to consume a light meal. After 15–20 min of rest, the participants' 10‐repetition maximum (10RM) in the bench press was determined. All participants completed session 1 in the same manner. Session 2 was conducted 1–2 weeks after Session 1, utilizing a same‐day crossover design (Figure 1), where participants were block randomized within their respective sex groups to either Protocol A or Protocol B. Prior to the execution of both protocols, EMG electrodes were applied, and a maximum voluntary isometric contraction (MVIC) was performed to collect muscle activation data for the TB, PM, and DA muscles during the two protocols.

**FIGURE 1:**
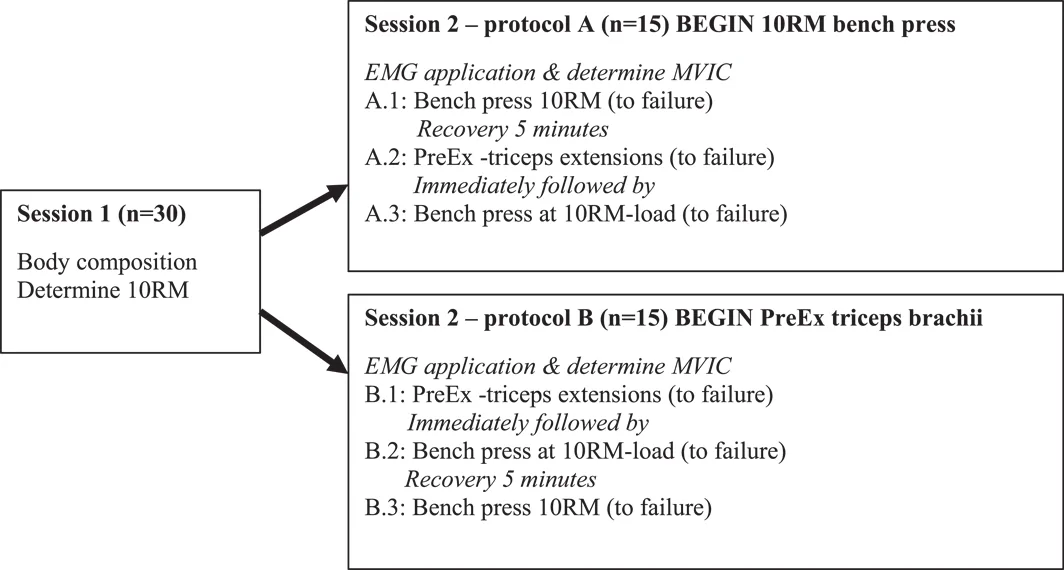
Experimental design for the different pre‐exhaustion (PreEx) protocols.

Protocols A and B consisted of the same exercises, but the timing of the PreEx method differed between the two. Protocol A commenced with a 10RM bench press to failure using the 10RM weight determined during Session 1. This was followed by a 5‐minute recovery period. Subsequently, PreEx of the TB to failure was performed, immediately followed by another bench press with the same 10RM load until failure. In Protocol B, the sequence was identical to Protocol A, but the order of PreEx was reversed. Participants began with PreEx of TB to failure, followed by the 10RM bench press from Session 1. After a 5‐minute recovery, the 10RM bench press was repeated. The 5‐min recovery period was selected based on previous recommendations for PreEx protocols (Gołaś et al. 2017). In both protocols, the PreEx method consisted of standing overhead triceps extensions with a weight plate (ELEIKO sport, Halmstad, Sweden) until they reached muscular failure. Weight was selected based on the participant's strength with the goal of reaching failure at approximately 15–20 repetitions. The appropriate load was determined during session 1. The participant stood next to the bench, allowing them to assume the starting position and begin the bench press immediately after reaching failure, thereby minimizing recovery time, as recommended (Jones 1970). The participants were asked to refrain from any upper body exercises 48 hours before sessions 1 and 2 and avoid caffeine and nicotine 4 h before the sessions.

#### 2.2.1 Assessment of the 10RM Bench Press

The 10RM bench press assessment session began with a warm‐up consisting of 12 repetitions at 50% of the participant's own estimated 10RM maximum load in the bench press. The starting position for the bench press test was lying supine on a bench with the arms held straight, feet were firmly on the floor and with gluteus maximus, the lower back, upper back and head firmly planted on the bench with elbows fully extended. The bench press was performed following the guidelines established by the National Strength and Conditioning Association (Haff and Triplett 2016), with a set velocity of 2 seconds for both the concentric and eccentric phases, paced by a metronome. Participants were instructed to lift the barbell off the rack and maintain full elbow extension before initiating the movement. Upon starting the metronome, they were required to eccentrically lower the bar until it made contact with the mid‐chest, after which they concentrically raised the bar to full elbow extension for each repetition. To ensure consistency in technique, participants were instructed to maintain the same hand placement throughout all trials using a personalized grip width corresponding to 165% of their biacromial breadth (Simpson et al. 1997). The 10RM test was performed with a 20 kg Olympic barbell with weight plates (ELEIKO sport, Halmstad, Sweden), and the starting load for the 10RM assessment session was based on the participant's own estimation. The weight was gradually increased with load increments of 0.25–5 kg, until participants reached the maximum load they could lift for 10 consecutive repetitions without exceeding this number, maintaining a constant velocity of 4 seconds per repetition, as described. Test leaders served as spotters, assisting participants in determining 10RM by adding incremental weights to the barbell and providing verbal encouragement throughout the testing process. The assessment of 10RM was performed using 5‐min resting periods between trials (Brennecke et al. 2009).

#### 2.2.2 Electromyography and Assessment of Muscle Activity

For the assessment of surface muscle activity, EMG‐electrodes were attached during the preparation for session 2, protocol A or B. First, the skin was shaved if needed and cleaned with alcohol. Second, single‐use silver/silver chloride surface electrodes (Ag/AgCl, Blue M‐00‐S, Ambu A/S, Ballerup, Denmark) with an inter‐electrode distance of 2 cm were attached on the right side of the body to the PM, the long head of the TB, and the DA. The measurement electrodes were placed parallel to the muscle fibers and the reference electrode was placed perpendicular to the measurement electrode. The exact position of the electrodes on each muscle followed the recommendation by SENIAM (http://www.seniam.org). Muscle activity was measured with the MEGA system (Mega WBA, MEGA Electronics Ltd, Kuopio, Finland) and its software MegaWin 3.1 at a sampling rate of 1000 Hz amplified. The EMG signal was processed with an average root mean square algorithm using a 100 ms moving average window. The EMG data analysis used the average values from three repetitions (the second to the fourth repetition). In cases where a participant was unable to complete four repetitions after the PreEx, the average was calculated from the second to the final repetition. Lastly, all values were normalized to maximal voluntary isometric contraction (MVIC) and expressed as a percentage of MVIC (%MVIC).

MVIC tests were performed in a bench press position pressing an immobilized barbell in a position where the elbow joint was in a 90° of flexion and the shoulders in 90° of abduction. The test protocol involved three trials of MVIC, each held for 5 seconds. The peak values from these trials were averaged and used as a reference for the muscles' 100% activation levels (Konrad 2006). A consistent exercise was employed for the MVIC across all three muscles, as recommended by Soares et al. (2016).

### 2.3 Statistical Analysis

All data were analyzed in SPSS (v. 28, IBM SPSS Statistics, IBM Corporation, Armonk, US) and are reported as mean ± standard deviation (SD). Based on EMG results from Brennecke et al. (2009), who found a medium effect size (ES) in TB with and without PreEx, we calculated a priori sample size (*α* = 0.05, *β* = 0.80) with a medium ES (Cohen's *f* = 0.25) for a 2*2 ANOVA. Results estimated that 34 participants were needed (using the G*Power program). Due to difficulties recruiting female resistance trained participants, only 30 participants (15 women) were included in total. Independent t‐tests were used for analyses of sex‐differences in body composition, relative strength and magnitude of muscle activation at baseline. A mixed model analysis of variance (ANOVA) was used with a 2 intervention (without PreEx/with PreEx) * 2 sex (women/men) design. If main effects (ME) or interaction (INT) showed significance at *p* < 0.05, subsequent post‐host‐hoc analyses (Bonferroni‐adjusted t‐tests) were performed. The ES based on 2*2 ANOVA was determined with the partial eta‐squared ($\eta_{p}^{2}$), and interpreted based on the following cut‐offs: 0.01 as small ES, 0.06 as medium ES and 0.14 and above as large ES (Maher et al. 2013). Moreover, Hedge's g pairwise ES was performed and interpreted based on the following cut‐offs: 0.20 as small, 0.50 as medium and 0.80 as large (Lakens 2013). The level of significance was set at *p* < 0.05 for all analyses.

## 3 Results

No difference was observed in age (*p* = 0.101) between the women and men included in the study. As anticipated, women were shorter, lighter, and exhibited greater fat mass but lower skeletal muscle mass and fat‐free mass (*p* ≤ 0.001) compared to men, whereas no significant difference in BMI was observed between the sexes (*p* = 0.072) (Table 1).

**TABLE 1:** Body composition and 10 repetition maximum (10RM) bench press load in women and men, including number of repetitions lifted without pre‐exhaustion (PreEx) and after PreEx of the triceps brachii before subsequent 10RM bench press.

	Women (*n* = 15)	Men (*n* = 15)	*t*‐test *p*‐value
Age (years)	26.5 ± 4.2	24.3 ± 3.0	
Body mass (kg)	66.4 ± 10.0	86.1 ± 10.0	
Height (cm)	165.7 ± 5.6	182.4 ± 8.1	
Skeletal muscle mass (kg)	28.0 ± 3.1	45.6 ± 10.1	< 0.001
Fat free Mass (FFM) (kg)	42.9 ± 6.5	72.8 ± 9.4	< 0.001
Body fat (%)	23.5 ± 8.9	13.3 ± 4.5	< 0.001
Body mass index	24.1 ± 3.1	25.8 ± 1.6	0.072
10RM load (kg)	37.1 ± 6.5	64.0 ± 7.1	< 0.001
10RM load/body mass (%)	57.3 ± 14.1	74.7 ± 7.9	< 0.001
10RM load/FFM (%)	87.5 ± 17.2	88.6 ± 9.9	0.824
Repetitions without PreEx	10	10	
Repetitions after PreEx	4.3 ± 2.6	3.8 ± 2.2	0.546

The average load lifted during 10RM was 37 kg for women and 64 kg for men (*p* < 0.001; Table 1). In this study, the 10RM load relative to body mass was still higher for men than women (*p* < 0.001), but when the 10RM load was related to FFM, the sex‐difference disappeared, with women lifting 87.5% of their FFM and men 88.9% of their FFM (Table 1). In addition, after PreEx, the number of repetitions lifted in the bench press (at the same load as 10RM) was lower for both women and men in a similar fashion (*p* = 0.55) going from 10 repetitions without PreEx to 4.3 ± 2.6 (women) and 3.8 ± 2.2 (men) repetitions after PreEx (Table 1).

### 3.1 Electromyographic Activity With or Without Pre‐Exhaustion

After PreEx, the muscle activation in PM was significantly increased (ME_intervention_ *p* < 0.001; $\eta_{p}^{2}$ = 0.558) during the ensuing bench press compared to the 10RM‐bench press in both women (post‐hoc *p* < 0.001; *g* = −1.08) and men (post‐hoc *p* = 0.002; *g* = −1.05), as shown in Figure 2. However, no interaction (INT *p* = 0.315; $\eta_{p}^{2}$ = 0.036) or sex difference (MEsex *p* = 0.456; $\eta_{p}^{2}$ = 0.020) for muscle activation in PM was found. Comparison of muscle activation in TB with and without PreEx showed a significant interaction (INT *p* = 0.023; $\eta_{p}^{2}$ = 0.170) indicating that PreEx before bench press elicited a significant increase in TB muscle activation for women (post‐hoc *p* = 0.020; *g* = −0.50) but not for men (post‐hoc *p* = 0.358; *g* = 0.34) compared to 10RM‐bench press (Figure 2). DA muscle activation during bench press showed no differences (INT *p* = 0.776; $\eta_{p}^{2}$ = 0.003) in neither women nor men (ME_sex_ *p* = 0.725; $\eta_{p}^{2}$ = 0.005), nor with or without PreEx (ME_intervention_ *p* = 0.074; $\eta_{p}^{2}$ = 0.110) as shown in Figure 2.

**FIGURE 2:**
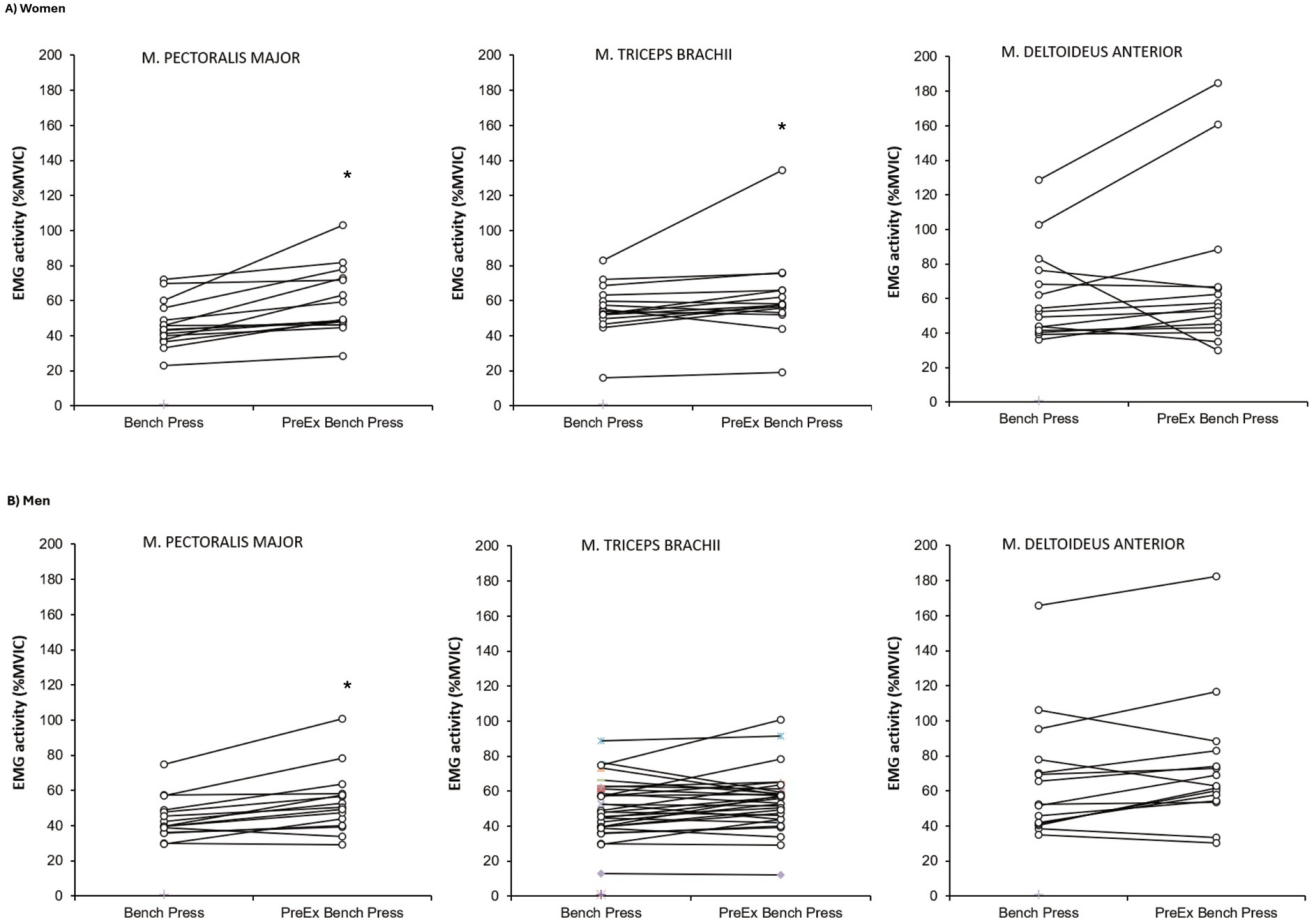
Comparison of electromyography (EMG) muscle activity between 10RM bench press without pre‐exhaustion (PreEx) and 10RM bench press with PreEx of triceps brachii immediately before 10RM bench press for women (A) and men (B) in muscles pectoralis major, triceps brachii and deltoideus anterior. %MVIC = muscle activity as percent of maximum voluntary isometric contraction. 10RM = 10 repetition maximum. *Significantly different from 10RM bench press without PreEx (*p* < 0.05).

Another secondary sex difference examined in this study, not related to PreEx, was whether the magnitude of EMG muscle activity during the bench press without PreEx differed between men and women. As indicated by the ANOVA analyses, no sex differences were observed in any of the muscles investigated during the bench press only condition. Additionally, independent t‐tests comparing %MVIC muscle activation between women and men during bench press without PreEx confirmed these findings (PM *p* = 0.63; TB *p* = 0.69; DA *p* = 0.61).

## 4 Discussion

Several important findings emerged from the present investigation. First, a sex difference was observed in muscle activation during the bench press: PreEx prior to the bench press led to increased TB activation in women but not in men. Additionally, both women and men demonstrated similar increases in PM activation following PreEx, whereas no changes in DA activation were detected in either sex. Furthermore, no sex differences were identified in the number of repetitions completed after PreEx or in the magnitude of EMG activation during the bench press without PreEx.

In the present study, muscle activation in PM, TB and DA was measured during bench press with or without a preceding PreEx. The results for men and women showed higher muscle activation in PM during the bench press after PreEx. These findings align with previous studies conducted on men only (Guarascio et al. 2016; Soares et al. 2016) but contrast with Gołaś et al. (2017), who reported no increase in PM muscle activity following PreEx of the TB in men. No previous research has investigated PreEx and EMG muscle activation in the bench press among women. We found that women had an increase in TB muscle activation after PreEx, a result that is somewhat counterintuitive in that an increase in PM and a decrease (or no change) in TB would have been expected based on previous studies done on men (Guarascio et al. 2016; Soares et al. 2016). Our results for PM showed that after PreEx, PM muscle activity increased during the subsequent bench press in both sexes. This result was more expected since it is assumed that PM will compensate for the fatigued TB so that the exercise could continue for a little longer (Guarascio et al. 2016; Soares et al. 2016). One possible explanation for the increased TB activation observed in women following PreEx could be a sex specific neuromuscular strategy under fatigued conditions, whereby women may rely more on synergistic muscles to maintain task performance. Differences in relative strength, fatigue resistance, or motor unit recruitment patterns between sexes may contribute to this response (Gentil et al. 2007; Hicks et al. 2001; Hunter 2014). However, no sex differences were observed in the number of repetitions performed after PreEx (from 10 to 4 repetitions after PreEx in both women and men), which does not support the presence of differences in TB muscle fatigue. As previous PreEx studies with EMG in the bench press have been conducted exclusively in men, it remains unclear whether these findings reflect true sex‐based differences or task‐specific adaptations, warranting further investigation.

As mentioned, the effects of PreEx on sex‐differences during the bench press have not yet been investigated; however, sex‐differences in EMG activity during the bench press (without PreEx) had been investigated previously. We found no sex‐differences in average EMG activation (%MVIC) between women and men in any of the three muscles in the 10RM bench press. In contrast, Alizadeh et al. (2020) investigated sex‐differences in muscle activation in the bench press and found that PM EMG activity in the concentric phase was higher in women compared to men but saw no differences in TB or DA (eccentric phase). Results from another study comparing changes in EMG activation from 55% to 100% of 1RM indicated that women and men had differences in maximal muscle activation depending on muscle and load; however, no clear conclusion could be drawn due to the inclusion of only five women (Gołaś et al. 2017). In a more recent study, Mausehund and Krosshaug (2023) observed that women had higher EMG mean activation in the TB long head, and lower EMG mean activation in the PM compared to men, with no differences in DA. It is difficult to draw any main conclusions since results from these studies show somewhat opposite results, they analyzed EMG differently (peak vs. mean values, concentric/eccentric vs. whole movement) and one study didn't normalize to %MVIC.

A strength of this study is its novel comparison of resistance‐trained women and men performing PreEx in the bench press, an area that has not been previously investigated. Other researchers have chosen a crossover design on separate days, but we see it as a strength that the participants did both bench press sessions (with and without PreEx) with EMG on the same day. When EMG studies on a bench press with and without PreEx are conducted on different days (Gołaś et al. 2017; Guarascio et al. 2016), the EMG electrodes must be removed and reset, which can introduce more noise in the measurements. To counteract any possible effects of PreEx on the 10RM bench press performed without PreEx, we employed a same‐day crossover design where half of the participants started with 10RM bench press only (protocol A) and the other half started with PreEx of TB before bench press (protocol B).

There were several limitations to the study. Recruiting 34 participants proved challenging, particularly finding women with resistance training experience who were willing to undergo palpation and electrode placement on the pectoralis major. Consequently, the target sample size of 17 participants per group was not reached, which may have reduced the statistical power to detect within‐ and between‐group differences. Moreover, EMG average muscle activation was calculated from repetitions two to four. For the participants who quickly fatigued following the PreEx protocol, the average activation was based on only one or two repetitions. This likely increased within‐participant variability; however, excluding these participants would instead have biased the results toward individuals with greater fatigue resistance. Another limitation was that participants were not matched for relative upper‐body strength. Consequently, differences in relative strength may have influenced the neuromuscular demands imposed by the pre‐exhaustion protocol and should therefore be considered when interpreting the findings. However, although men lifted greater absolute loads, the sex difference disappeared when the 10RM load was expressed relative to fat‐free mass, suggesting comparable strength relative to muscle mass between groups.

## 5 Conclusion

In women, PreEx of the TB led to increased subsequent muscle activation in both the TB and PM during the bench press, whereas in men, increased activation was observed only in the PM. PreEx also reduced the number of bench press repetitions similarly in both women and men. Thus, PreEx can enhance primary muscle activation during the bench press in both sexes, aiding hypertrophy and neuromuscular goals. However, women may show greater tricep compensation, requiring tailored volume and recovery. With no sex‐differences in traditional bench press activation, standard protocols can be applied when adjusted for strength and body composition.

## Funding

The study was financially supported by Halmstad University, Sweden.

## Ethics Statement

Ethics approval was obtained for this project from the Regional Ethical Review Board in Lund, Sweden (Dnr 2013/707).

## Conflicts of Interest

The authors declare no conflicts of interest.

## Data Availability

The data that support the findings of this study are available on request from the corresponding author. The data are not publicly available due to privacy or ethical restrictions.
